# The *Lichenophanes* Lesne, 1899 of the Palaearctic and Oriental Regions (Coleoptera, Bostrichidae) †

**DOI:** 10.3390/insects16040411

**Published:** 2025-04-14

**Authors:** Jerzy Borowski, Hervé Brustel, Artur Rutkiewicz, Tomasz Oszako, Andrzej Lasoń

**Affiliations:** 1 Department of Forest Protection, Institute of Forest Sciences, Warsaw University of Life Sciences SGGW, ul. Nowoursynowska 159/34, 02-766 Warsaw, Poland; artur_rutkiewicz@sggw.edu.pl; 2 Université de Toulouse Ecole d’ingénieurs de Purpan, UMR 1201 Dynafor, 75 voie du TOEC BP 57611, 31076 Toulouse, Cedex 3, France; herve.brustel@purpan.fr; 3 Faculty of Civil and Environmental Engineering, Białystok University of Technology, Wiejska 45 A, 15-351 Białystok, Poland; t.oszako@pb.edu.pl; 4 Independent Researchers, ul. Wiejska 4B/85, 15-352 Białystok, Poland; haptos@interia.pl

**Keywords:** horned powderpost beetles, Bostrichini, new species, new subspecies, new status, key for identification, geographical distribution, Asia, Europe, North Africa

## Abstract

This paper includes a review of the Palaearctic and Oriental species of the genus *Lichenophanes* Lesne, 1899. It contains a description of the new species, *Lichenophanes juxtaorientalis* n. sp., which occurs in Cyprus and Crete. A new subspecies, *Lichenophanes varius turanicus* n. subsp.—occurring in Crete, Cyprus, and the Middle East—was separated from the species *Lichenophanes varius* (Illiger) and described. Moreover, this paper characterizes *L. carinatus* (Lewis) n. stat., which had been treated as a synonym of *L. carinipennis* (Lewis). Two maps present the geographic distribution of particular species, and the final part of this paper contains keys to identify them.

## Simple Summary

This paper presents a description of a new Western-Palaearctic species of beetles of the horned powderpost beetle family. The new species is found in the eastern part of the Mediterranean subregion and has been identified in Crete, Cyprus, and also the eastern part of Syria. Morphological analyses showed that the specimens of *L. varius* occurring in Crete, Cyprus, and the Middle East significantly differ from the specimens inhabiting the continental part of Europe. In connection with the above, it was suggested that a new subspecies be created for *L. varius*. Also, while analyzing the morphology of *L. carinipennis* specimens from East Asia, some differences were noted between those coming from the Oriental and those from the Palaearctic parts. In this case, tropical specimens were reinstated as a separate species. To sum up, in the Western Palaearctic, there are three species and one subspecies; in the Eastern Palaearctic, there is one species; and in the Oriental area, there is also one species of the genus *Lichenophanes*. All the species are presented in color macrophotographs, and maps showing the distribution of particular species are also included. The final part of this paper includes keys for the identification of the studied species.

## 1. Introduction

The genus *Lichenophanes* was created by the French entomologist Pierre Lesne. It was described in the third volume of the world revision of horned powderpost beetles [1]. In addition to a description of the genus, the author also included descriptions of 24 species of the genus in question. The species described by Lesne included three Palaearctic ones, namely *L. carinipennis* (Lewis, 1896), *L. numida* Lesne, 1899, and *L. varius* (Illiger, 1801). The paper also covered the only Oriental species: *L. khmerensis* (Lesne, 1896). Nearly forty years later, a world catalog of horned powderpost beetles was published, also authored by P. Lesne [2]. In the catalog, he provided information about thirty-seven species of the genus *Lichenophanes*, while Palaearctic and Oriental regions were only represented by three of them: *L. carinipennis*, *L. numida*, and *L. varius*. The above-mentioned representative of the Oriental region, *L. khmerensis*, was included as a synonym of *L. carinipennis*. In 2007, volume 4 of the *Catalogue of Palaearctic Coleoptera* was published with the same three species presented again [3]. These data were also repeated in the *World Catalogue of Bostrichidae* [4]; however, it contained data of 41 species distributed on almost all continents. Below, we present a description of a new West-Palaearctic species of the genus *Lichenophanes* and a new subspecies of *L. varius*. Moreover, taxonomical changes were made within the East Asian species of the discussed genus. Part two of this paper contains keys to identify species as well as information on the geographical distribution and bionomy of particular species.

## 2. Materials and Methods

This paper was based both on published resources and on original material from European, Asian, and American museums and scientific institutions that store entomological collections.

Type material was analyzed at the Muséum National d’Histoire Naturelle, Paris (MNHN), and the Natural History Museum, London (MHNUK). Specimens of the new species and subspecies described below are deposited in the collection of the Department of Forest Protection at Warsaw University of Life Sciences (DFPW), Hervé Brustel’s private collection (HBPC), and at Andrzej Lasoń’s private collection (ALPC).

Color photos were taken with a Leica 205C stereoscopic microscope (Leica Microsystems GmbH, Wetzlar, Germany, Europe) equipped with a camera and photo-processing software. SEM photos were taken at the Institute of Zoology, Polish Academy of Science (PAN), Warsaw, Poland.

## 3. Results

### 3.1. General Information on the Genus Lichenophanes Lesne, 1899

Type species: *Bostrichus tristis* Fåhraeus, 1871: 669 [5], designation by Chûjô, 1937: 44 [6].

**Figure 1:**
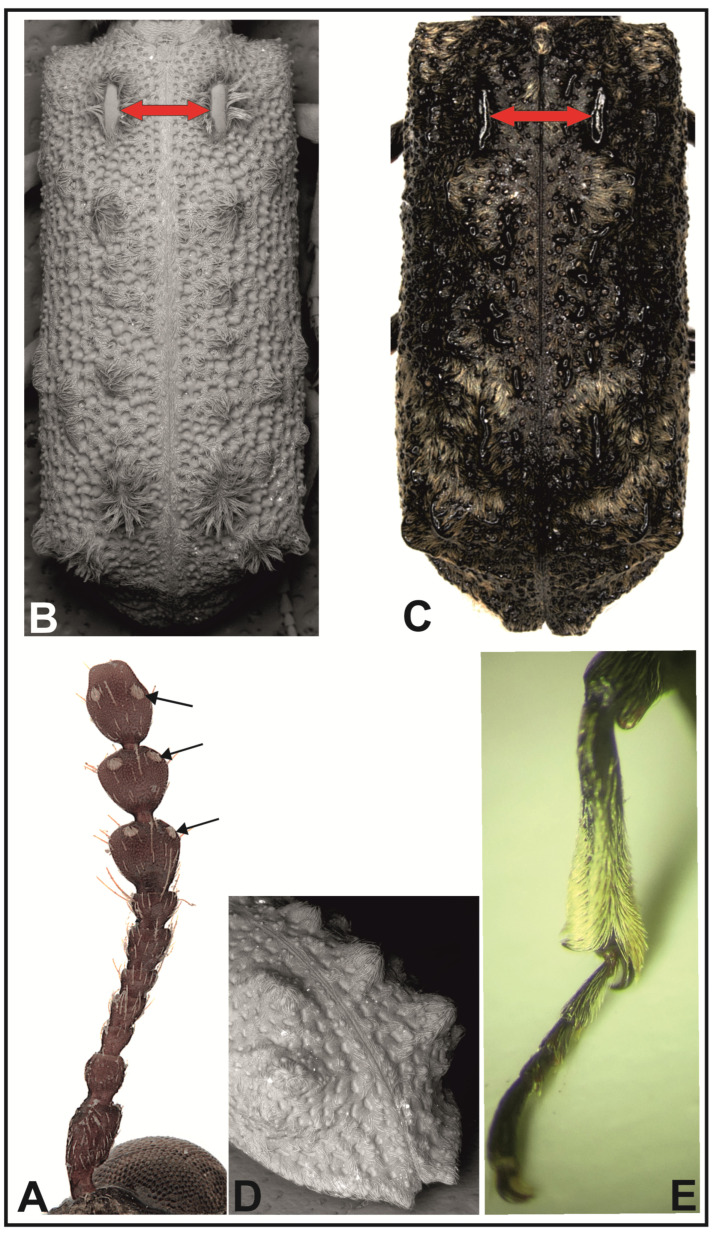
Elements of the morphological structure of adult individuals of *Lichenophanes* Lesne: (**A**) antenna of *Lichenophanes varius* (Illig.), where arrows show the sensory hollows; (**B**) surface of elytra of *Lichenophanes marmoratus* Lesne, where red arrows show the location of the parascutellar carinae of elytra; (**C**) surface of elytra of *Lichenophanes bedeli* (Lesne), where red arrows show the location of the parascutellar carinae of elytra; (**D**) elytral declivity and apex of elytra of *Lichenophanes verrucosus* (Gorham), semilateral view; (**E**) front tibia of *Lichenophanes bedeli* (Lesne).

Description: Body elongated, cylindrical, usually of cryptic coloration. Antennae short, with 10 antennomeres, ending with a three-segmented club; segments of the club tiny, usually strongly protuberant, with a pair of oval sensory hollows covered with golden setae (Figure 1A); pronotum and elytra in most species are densely covered with narrow, light, scale-like setae; additionally, various kinds of protuberant carinae and bigger and smaller calli occur on elytra. The most characteristic feature of the genus is a pair of short protuberant carinae in the anterior part of elytra (parascutellar carinae) (Figure 1B,C). Elytral declivity is not as clear as in, e.g., representatives of Xyloperthini or Apatinae, evenly sloping, usually convex all over the surface, and almost always without protruding denticles or calli. In some species, the apices of elytra may have characteristic tips (Figure 1D); in others, the tibia may be covered with dense scale-like setae (Figure 1E). The genus is characterized by a lack of visible sexual dimorphism. Its species are nocturnal, fly well, and are attracted to artificial light sources. During the day, the beetles hide on host trees as their characteristic coloring perfectly camouflages them in bark cracks or wood slots. Adults may also hide in holes left by the insects that have left the tree and under protruding bark. The beetles develop mainly in the trunks and branches of dead trees, and larvae feed on hardwood in the initial stage of fungi-caused decomposition.

### 3.2. Diagnosis, Description, and Biology of the New Species

**Figure 2:**
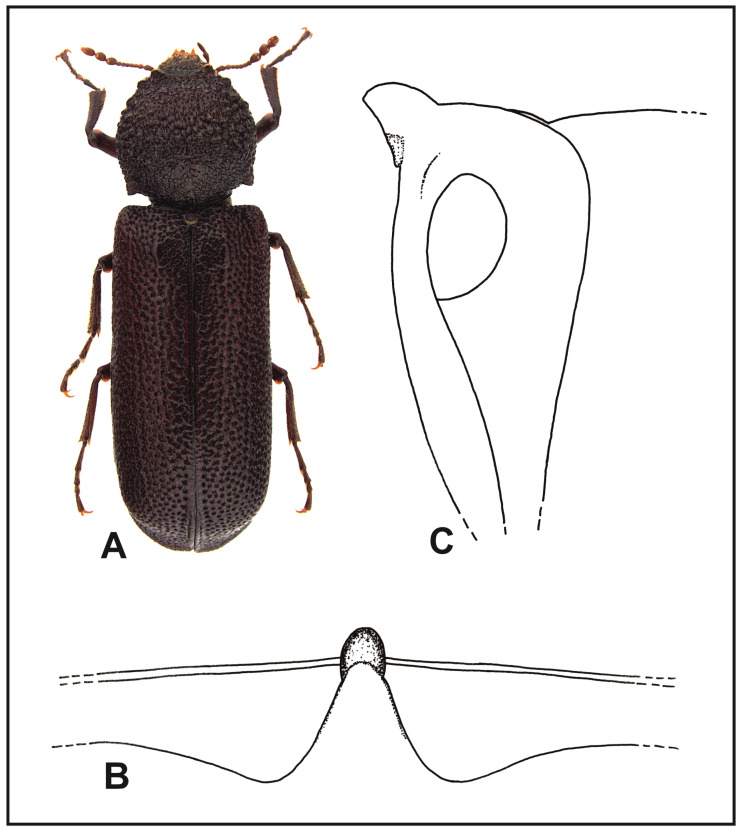
*Lichenophanes juxtaorientalis* n. sp.: (**A**) body, dorsal view; (**B**) intercoxal processes of the first abdominal sternite, dorsal view; (**C**) intercoxal processes of the first abdominal sternite, lateral view.

*Lichenophanes juxtaorientalis* Borowski and Brustel n. sp. (Figure 2A).

Type material and other information. **Holotype**: “Chypre, Miliou–Paphos, N34°56′16″, E32°27′50″, 21–28 VI 2012, lampe U.V., leg. G. Miessen” (DFPW). **Paratypes**: same data as holotype, 1 ex. (HBPC); “O. Cyprus, Miliou–Paphos distr., 16–22 VI 2016, leg. G. Miessen”, 2 exx. (HBPC and DFPW); “Crète, Greece, Kolimvari, Deliana gorge, 6 VII 2011, lampe U.V., leg. G. Miessen”, 1 ex. (HBPC); “Crète, Rousakiana, 17 VI 2005, 830 m, leg. Peslier” 2 exx. (HBPC and DFPW); “Syria, Prov. Latakia, Salah Addin citadel, *Quercus*-plane, 6 VI 2010, leg. A. Kotán, E. Mizsei, T. Németh, N. Rahmé”, 1 ex. (HBPC).

Diagnosis: Thanks to the dark coloration of the body, scarce and unclear setation of the elytra, and poorly developed parascutellar carinae, the new species can be easily differentiated from all other known species of the Palaearctic and Oriental regions. At first glance, *L. juxtaorientalis* n. sp. may look similar in body shape and color to the species of the genus *Apate* Fabricius, 1775, *Phonapate* Lesne, 1895, or even *Xylomedes* Lesne, 1902; however, they have a different morphology of the head, particularly regarding the antennae, which have transversely elongated segments of clubs. The new species is also slightly similar to the dark-colored variety of *Bostrichus capucinus* (Linnaeus, 1758), which differs from it mainly in the morphology of the abdomen; the new species has very pronounced pleura sutures on the sides of abdomen segments, while in *B. capucinus,* the sutures are only slightly marked on the sides of the last abdominal segment. Additionally, in *L. juxtaorientalis* n. sp., the intercoxal process of the first abdominal sternite is widened and has lateral margins that connect by a sharp ridge with the pleural sutures of abdominal sides; in *B. capucinus,* the process is very narrow and has no lateral margins forming a ridge that would connect it with the abdominal sides.

Description: Length, 9.5–14 mm. Body elongated cylindrically, black–tawny to black. Labrum margin is slightly arched, with dense golden setae. Anterior margin of the clypeus is straight; the clypeus sides are rounded. The surface of the clypeus has small punctures, covered with recumbent, yellow–gray setae. The margin separating frons from clypeus is clearly visible along its width. Frons with punctures and short recumbent setae. The vertex and occiput part of the head are densely covered with small papillae. Eyes are small but considerably protuberant, clearly protruding beyond the head outline. Antennae of 10 antennomeres ending with three-segment clubs. Scape of the antennae is twice as long as the next two segments together. Pronotum is slightly wider than its length, rounded on sides; widest in its anterior part, right before the middle. Anterior angles of the pronotum are clear, in the form of bluntly sharpened calli. Anterior margin of the pronotum is arched, with short, forward-directed, single denticles on the sides. Sides of the anterior half of the pronotum are crenated; central and posterior parts of pronotum are covered with flattened papillae. Setae of pronotum are hardly visible, short, and recumbent on the anterior half of the pronotum, on its sides, posterior angles, and in the central part of the posterior margin. Scutellum convex, clearly protruding above the surface of the elytra and covered with yellow–gray, short setae. Humeral protuberances are visible. Parascutellar carinae of the elytra are poorly pronounced. Elytral puncturation is dense—punctures are deep and irregularly distributed. Surface of the elytra is nearly glabrous among punctures. Very tiny and recumbent setae are more visible on the sides and margins of the elytra, and also in the area of elytral declivity. The margins of elytral apices are thickened on the sides connecting with parallel margins of the declivity in the form of a small bump. Ventral part of the body red–tawny to black–tawny. Central suture of the metasternum is delicate, and disappears before the anterior process of the metasternum. Intercoxal process of the first abdominal sternite as in Figure 2B,C. Tibia in apical parts are slightly more densely covered with golden setae than in the basal and central parts.

Variation: apart from the body size and coloration, no morphological variation was observed between specimens from Crete, Cyprus, and Syria.

Etymology: the name of the new species is a word derived from Latin, *juxtaorientalis*—meaning Middle Eastern—because of its distribution.

Biology and phenology: Poorly known. Adults are active at night and fly in June and July to a source of artificial light together with the *L. varius turanicus* n. subsp.

### 3.3. Lichenophanes varius (Illiger, 1801)

*Apate varia* Illiger, 1801: 172 [7]
*Bostrichus Dufourii* Laterille, 1806: 7 [8]
*Apate gallica* Panzer, 1807: 17 [9]

The species was described in Germany (Hanover) by Illiger in 1801 [7]. Several years later, it was described again by Latreille [8] from France (Fontainebleau) as *Bostrichus doufourii*, and the name was synonymized by Jacquelin du Val (1861) [10]. Panzer, in 1807 [9], generally described *Apate gallica* from Germany, and this in turn was synonymized by Duftschmid in 1825 [11]. In 1899, Lesne [1] classified the species in the genus *Lichenophanes*.

*Lichenophanes varius* is a Western-Palaearctic species, connected mainly with the forest–steppe zone [12]. It occurs in Central and Southern Europe, reaching eastward to the Volga River and the Caspian Sea and south to Turkey, Syria, Iran, and Turkmenistan. It has also been found in Crete and Cyprus. Due to morphological differences in adults occurring in the south-eastern area of its range when compared to the forms from Western and Southern Europe, two subspecies have been differentiated and they are described below.

#### 3.3.1. Description, Biology, and Geographical Distribution of the Nominotypical Subspecies

**Figure 3:**
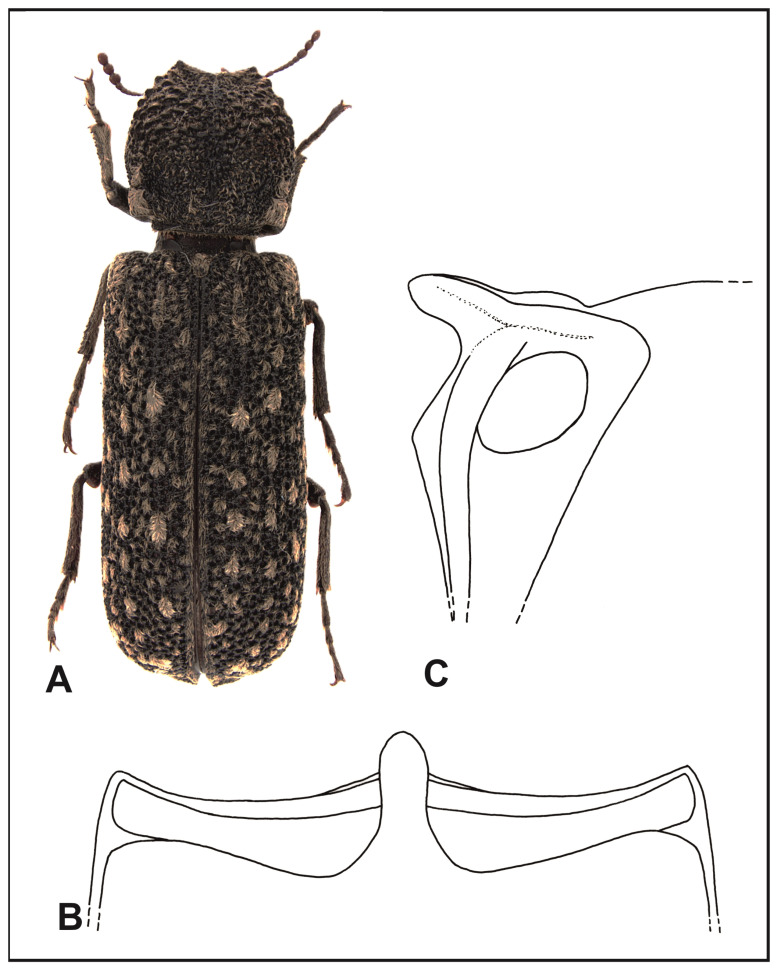
*Lichenophanes varius varius* (Illig.): (**A**) body, dorsal view; (**B**) intercoxal processes of the first abdominal sternite, dorsal view; (**C**) intercoxal processes of the first abdominal sternite, lateral view.

*Lichenophanes varius varius* (Illiger, 1801) (Figure 3A).

Description: Length, 5.5–13 mm. Cylindrical body, elongated, red–tawny to tawny. The margin of the labrum is straight or very slightly arched, with dense, golden setae. Anterior margin of the clypeus is straight; the sides of clypeus are rounded. Surface of the clypeus is densely covered with tiny punctures and covered with recumbent, yellow–gray setae. The margin separating the frons from clypeus is distinctly visible along the entire length. Frons and occiput of the head are covered with tiny papillae and recumbent setae. Eyes are small but very protruding, and clearly protrude beyond the head outline. Antennae of 10 antennomeres, ending with three-segmented clubs. The scape is as long as the next two antennomeres together. Pronotum is of similar length and width, rounded on the sides; the widest segment of the pronotum is in its anterior part, right before its middle. Posterior angles of the pronotum clear, in the form of sharply ending calli. Anterior margin of the pronotum is triangularly shaped, with upward protruding crenates on the sides. The anterior, middle, and sides of the pronotum are crenated; the posterior pronotum is covered with flattened papillae. Setation of the pronotum is visible, recumbent, in the form of narrow, scale-like setae; they are particularly dense in posterior angles, in the crenated area, and on the top and middle part of the posterior margin of the pronotum. Scutellum is elongated and convex, clearly protruding above elytra and densely covered with yellow–gray setae. Humeral protuberances are clearly visible. Parascutellar carinae of the elytra are clearly marked, strongly shining, and in young specimens (life-experienced) are almost completely covered with setae. Elytral punctures are dense, deep, and irregularly distributed. Surface of the elytra between punctures is covered with tiny, shining papillae. All the elytra have recumbent yellow–gray, scale-like setae, which form patches of various sizes. Sometimes the patches may form elongated, broken rows. Apical margins of the elytra are slightly thickened, jointly sharpened, and on the ventral side slightly dentated. The connection of the apical and lateral margins of elytral declivity is indistinct, often covered with setae. Ventral side of the body is red–tawny. Central suture of the metasternum is slight, in the form of an elongated, narrow line reaching the anterior process of the metasternum. Intercoxal process of the first abdominal sternite as in Figure 3B,C. Tibia in apical parts are slightly more densely covered with golden setae than on the remaining surface.

Biology and phenology: The subspecies is connected with various deciduous trees, but most often reported from oaks (*Quercus* spp.) and beeches (*Fagus* spp.). A compendium of information on the development of this species can be found in the paper by Nardi and Biscaccianti [13].

**Figure 4:**
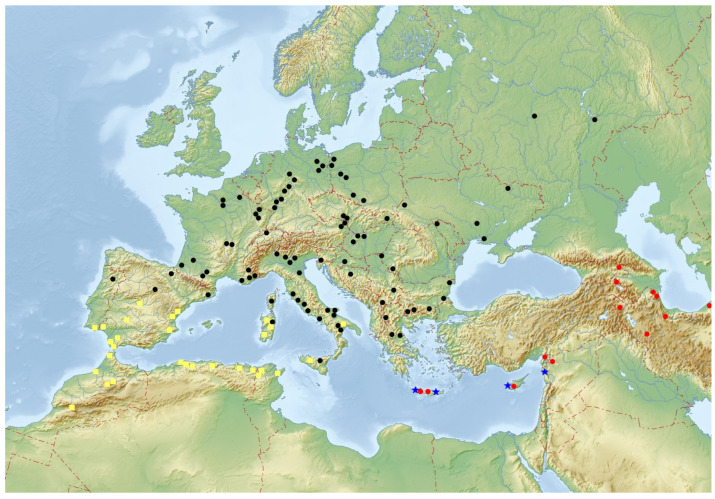
Geographical distribution of *Lichenophanes* species in the West Palaearctica; yellow square—*Lichenophanes numida* (Lesne); black dot—*Lichenophanes varius varius* (Illiger); red dot—*Lichenophanes varius turanicus* n. subsp.; blue star—*Lichenophanes juxtaorientalis* n. sp.

Distribution: Portugal, Spain, France (continental part), Corsica (France), Italy, Germany, Switzerland, Austria, Czechia, Poland, Slovakia, Ukraine, central and south Russia (European part), Hungary, Romania, Croatia, Bosnia and Herzegovina, Serbia, Bulgaria, Albania, Kosovo, and Greece (continental part) (Figure 4).

#### 3.3.2. Diagnosis, Description, Biology, and Zoological Distribution of the New Subspecies

**Figure 5:**
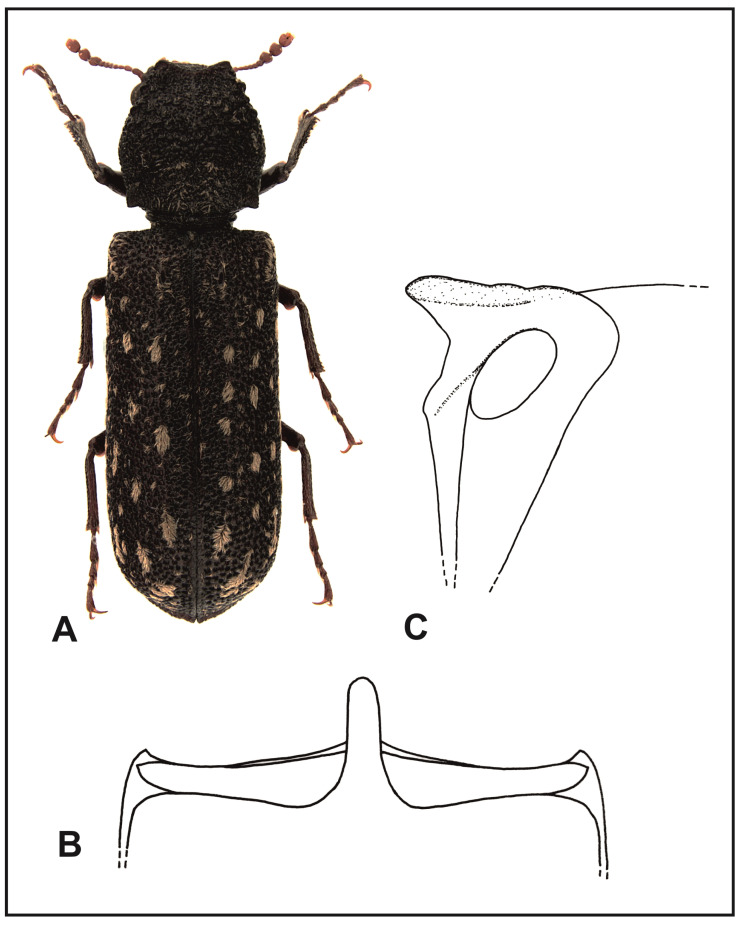
*Lichenophanes varius turanicus* n. subsp.: (**A**) body, dorsal view; (**B**) intercoxal processes of the first abdominal sternite, dorsal view; (**C**) intercoxal processes of the first abdominal sternite, lateral view.

*Lichenophanes varius turanicus* Borowski and Brustel n. subsp. (Figure 5A).

Type, material, and other features. **Holotype**: “Chypre (Paphos), Miliou, N34°56′16″, E32°27′50″, (lumieres U.V.), 24–29 VI 2006, 1 ex., 16-19 VI 2008, (207 m), leg. G. Miessen” (DFPW). **Paratypes**: “Iran, Golestan prov., ad Sadeh Abad, N37°22′06”, E55°40′45″, 23 V 2018, leg. M. Bidas” (DFPW); “Iran, Golestan prov., Golestan N.P., 45 km E Minudasht., N37°36, E55°93′, 900 m, 11 VI 2010, 1 ex., leg. Walter Grosser” (HBPC); “Iran, Golestan, Alma Gol, 2 km NW of Tengli, N37°26′, E54°38′, 16 VI 2015, 8 m, *Tamarix* sp., 1 ex., leg. A. Lasoń” (ALPC); “Iran bor, Golestan prov., 50 km E Minudashi, 9 km E Tangrah vil., VI 2017, 540 m, 2 exx., leg. V. Nový“ (DFPW); “Greece, Crete, Lakki, 3–15 VI 1999, 1 ex., leg. P. Moravec” (HBPC); “Greece, Crete, N35°14′, E25°27′, 6 V 2006, 590 m, oak woods, 1 ex., leg. A. Lasoń” (DFPW); “Chypre (Paphos), Miliou, N34°56′16″, E32°27′50″, (lumieres U.V.), 16–19 VI 2008, (207 m), 1 ex., 21–28 VI 2012, 1 ex., 2–8 VII 2012, 2 exx., 11–17 VII 2012, 1 ex., 16–12 VI 2016, 1 ex., leg. G. Miessen” (HBPC and DFPW); “Georgia, Kakheti, Vashlovani N.P., Kaklis Kure, N41°17′, E46°44′, 166 m, 15 VI 2021, decidous trees, 1 ex., leg. A. Lasoń” (DFPW); “Azerbaijan, N38°57′, E48°26′, 17 km NE of Yardimli (23 km by road), Yardimli rayonu, 28–31 V 2010, a = 368 m, 1 ex., leg. R. Królik” (DFPW); “Azerbaijan, Talysh Mts., Yardimli rayonu, 23 km NE of Yardimli, N38°56′, E48°26′, 368–450 m, oak woods, at light, 7 VI 2013, 1 ex., 4 VI 2013, 1 ex., leg. A. Lasoń” (ALPC); “Azerbaijan, Talysh Mts., Yardimli rayonu, 23 km NE of Yardimli, N38°56′, E48°26′, 368–450 m, *Quercus* sp. under bark, 30 V 2010, 1 ex., 27 V 2013, 29 V 2010, 1 ex., 30 V 2023, 1 ex., 2 VI 2013, 1 ex., 1 VII 2024, 1 ex., leg. A. Lasoń” (DFPW and ALPC); “Azerbaijan, Talysh Mts., Lenkoran rayonu, Hirkan N.P. ad Hirkan, N38°39′, E48°48′, 40 m, oak woods, 24 VI 2024, 1 ex., leg. A. Lasoń” (ALPC); “Azerbaijan, Talysh Mts., Lerik rayonu, 2 km NE of Peştətük, N38°46′, E48°34′, 372 m, 29 VI 2024, 1 ex., oak forest, leg. A. Lasoń” (ALPC); “Chypre, Miliou-Paphos, N34°56′16″, E32°27′50″, 21-28 VI 2012, 1 ex., 1/6 VI 2019, 1 ex., 23/29 VI 2019, 1 ex., 30 VI 2019, 1 ex., 26/31 V 2019, 3 exx., lampe U.V., leg. G. Miessen” (HBPC and DFPW).

Diagnosis: A new subspecies with a clearly thinner setation of elytra than *L. varius varius*; the setae are grouped into small, well-isolated patches and their pattern resembles the pattern of patches in *L. numida* more than in *L. varius varius*. Surface of the elytra between punctures in the new species is smooth, without tiny papillae, which on the other hand occur in the nominative subspecies. Also, the intercoxal process of the first abdominal sternite, when seen from above, is narrower with partly parallel sides, and at the apex, it is narrowly rounded; in *L. varius varius*, the process is wider and widely rounded at the apex. On the other hand, in *L. numida*, the intercoxal process is conical; also, the apical margins of the elytra are slightly flattened and elongated to the back. In both subspecies of *L. varius*, the apical margins of the elytra are evenly convex and are not elongated to the back.

Description: Length, 7.5–13 mm. Body cylindrical elongated, red–tawny to tawny. Margin of the labrum is straight or very slightly arched, with thick, gold setae. Anterior margin of the clypeus is straight; sides of clypeus are rounded. Surface of the clypeus is densely covered with tiny punctures and recumbent, yellow–gray setae. The margin separating the frons from the clypeus is distinctly visible along the entire length. Frons, vertex, and occiput are covered with tiny papillae and recumbent setae. Eyes are small but strongly protruding, clearly beyond the head outline. Antennae of 10 antennomeres ending with three-segmented clubs. Scape as long as the subsequent two segments together. Pronotum is of similar length and width, sides are rounded; the widest part of the pronotum is in its anterior part, right before its middle. Posterior angles of the pronotum are clearly marked, in the form of sharp-ended calli. Anterior margin of the pronotum is triangularly shaped, on the sides with upward protruding crenates. Anterior part, middle, and sides of pronotum are crenated; posterior part of the pronotum is covered with flattened papillae. Pronotum setation is clearly visible, recumbent, in the form of narrow, scale-like setae; the setae are particularly densely located in posterior angles, in the crenated area, and at the apex and middle part of the pronotum base. Scutellum is elongated, convex, clearly protruding above the surface of the elytra and densely covered with yellow–gray setae. Humeral protuberances are clearly visible. Parascutellar carinae are clearly marked, strongly shining, and covered with thin setae. Puncturation of the elytra is dense; punctures are deep and irregularly spaced. Surface of the elytra between the punctures is smooth without tiny, shining papillae. Elytra are with recumbent yellow–gray, scale-like setae, which form small patches. Sometimes the patches can form elongated, broken rows. Apical margins of the elytra are slightly thickened, jointly sharpened, on the ventral side with slight dentation. Connection of apical and parallel margins of elytral declivity is not clear. Ventral part of the body is red–tawny. Central suture of the metasternum is delicate, in the form of an elongated, narrow line reaching the anterior process of the metasternum. Intercoxal process of the first abdominal sternite as in Figure 5B,C. Tibia in apical parts are slightly more densely covered with golden setae than on the remaining areas.

Variation: specimens from Iran may have a slightly less developed parascutellar carinae than those from Crete, Cyprus, Georgia, or Azerbaijan.

Etymology: the subspecies is named after its distribution, which mainly covers the Irano-Turanian province of the Mediterranean subregion.

Biology and phenology: The subspecies has not been differentiated from *L. varius varius* up to now and probably some data on *L. varius varius* refer to *L. varius turanicus* n. subsp. Adults were caught in Cyprus and Crete near a light source in June. In Iran and Azerbaijan, the subspecies was caught in the last ten-day period of May, in June, and in early July.

Distribution: Crete (Greece), Cyprus, south-east Turkey, Syria, Iran, Azerbaijan, Georgia, and SW Turkmenistan (Figure 4).

### 3.4. Diagnosis, Description, and Biology of Lichenophanes numida Lesne, 1899 (Figure 6)

*Lichenophanes numida* Lesne, 1899: 478 [1].

**Figure 6:**
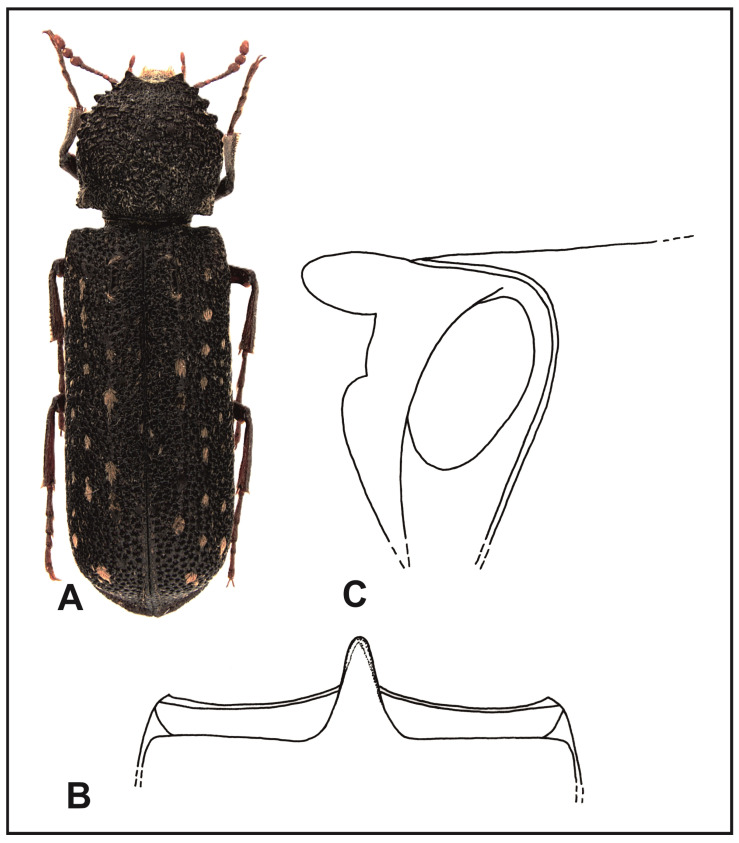
*Lichenophanes numida* Lesne: (**A**) body, dorsal view; (**B**) intercoxal processes of the first abdominal sternite, dorsal view; (**C**) intercoxal processes of the first abdominal sternite, lateral view.

Description: Body length, 7.8–14 mm. Body cylindrical and elongated, red–tawny to black–tawny. Labrum margin is nearly straight or very slightly arched, with dense, golden setae. Anterior margin of the clypeus is straight; clypeus sides are rounded. Surface of the clypeus with tiny papillae, covered with long, raised yellow–gray setae. The margin separating the frons from clypeus is clearly visible all along. Frons, vertex, and occiput are covered with tiny papillae and short, recumbent setae. Eyes are small but strongly protruding, clearly beyond the outline of the head. Antennae of 10 antennomeres, ending with three-segmented clubs. Scape is as long as the subsequent two segments together. Pronotum is slightly wider than long, rounded on the sides; the widest in its anterior part, right before its middle. Posterior angles of pronotum are clear, in the form of triangular, sharpened calli. Anterior part of pronotum is shallowly and archly emarginated, with tiny hook-like, forward-directed denticles on the side. Anterior part, middle, and sides of pronotum are crenated; posterior part of pronotum, particularly in the area of posterior angles, are covered with papillae. Setation of pronotum is recumbent, in the form of narrow, yellow–gray setae, which are scarce and do not completely cover pronotum surface in any place. Scutellum is elongated, convex, clearly protruding above the surface of elytra, and densely covered with yellow–gray setae. Humeral protuberances are poorly marked. Parascutellar carinae are clearly marked, strongly shining, with scarce setae. Elytral puncturation is dense; punctures are deep and irregularly spaced. Surface of the elytra between punctures is smooth without tiny, shining papillae. Elytra with recumbent yellow–gray setae, which form small patches. Sometimes the patches can form elongated, broken rows. Apical margins of elytra are slightly thickened, jointly sharpened, on the ventral side slightly crenated. Connection between apical and parallel margins of elytral declivity is clearly marked. Ventral part of the body is red–tawny. Central suture of metasternum is delicate in the form of an elongated, narrow line reaching just beyond the middle of the metasternum length. Intercoxal process of the first abdominal sternite as in Figure 6B,C. Tibia in apical parts are slightly more densely covered with golden setae than on the remaining surface.

Biology and phenology: A polyphagous species. It feeds on dead trunks and branches of various tree species, e.g., *Eucalyptus globulus* Labill. In the Atlas Mountains, it is found up to 2000 m above sea level [14].

It has been described from Morocco (Tanger) and Algeria (area of Algiers, Dra-el-Mizan, Fort National (=Larbaa Nath Irathen), Robertville (=Emdjez Edchich), and La Calle (=Annaba)).

Distribution: Europe: Portugal, Spain, and Italy (Calabria, Sardinia, and Sicily); N. Africa: Morocco, Algeria, and Tunisia (Figure 4).

### 3.5. Description, Biology, and Geographical Distribution of Lichenophanes carinatus (Lewis, 1896) Stat. Rev. (Figure 7A)

*Apate carinata* Lewis, 1896: 339 [15]
*Bostrychus khmerensis* Lesne, 1896: 511 [16]

Type material: **Holotype** of *Apate carinata*: “Andaman Isles”, “*Apate carinata* Lewis Type”, “Museum Paris, Coll. H.-S. Gorham 1911” (MNHN). **Syntype** of *Bostrychus khmerensis*: “Pnom-Penh, Cambodja, Fleutiaux” (MNHN).

**Figure 7:**
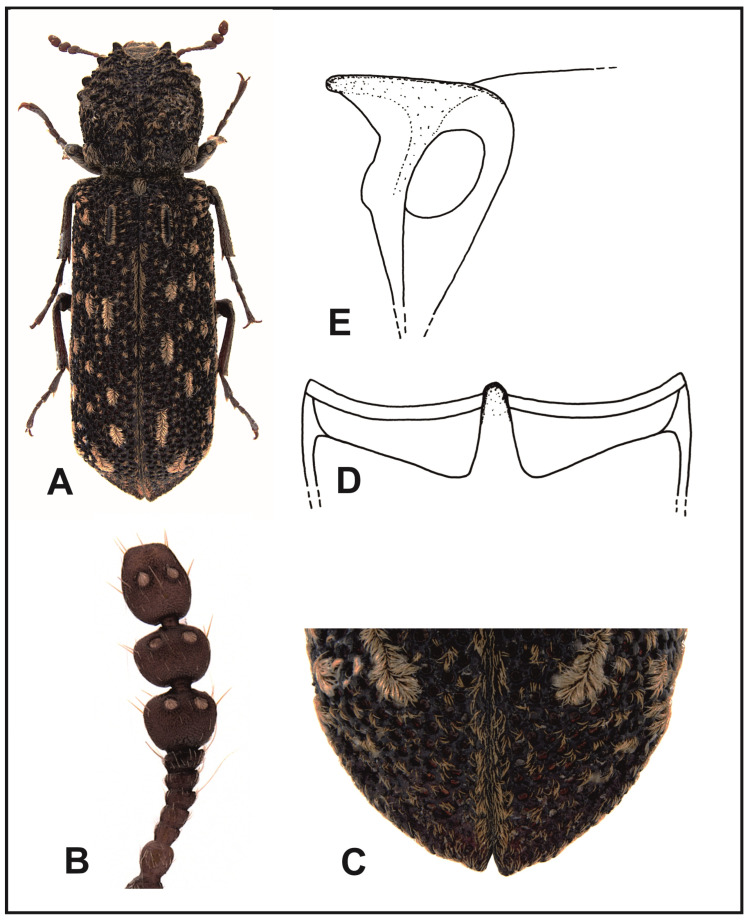
*Lichenophanes carinatus* (Lewis): (**A**) body, dorsal view; (**B**) antenna; (**C**) apex of elytra; (**D**) intercoxal processes of the first abdominal sternite, dorsal view; (**E**) intercoxal processes of the first abdominal sternite, lateral view.

Description: Body length, 8.5–11.5 mm. Body cylindrical, elongated, red–tawny to dark–tawny. The anterior margin of labrum is shallowly arched, with densely spaced golden setae. The anterior margin of the clypeus is nearly straight. Clypeus covered with slightly raised, dense, yellow–gray setae. The suture separating the clypeus from frons is clearly visible only in its central part. Frons with papillae, densely covered with golden, recumbent setae. Vertex and occiput densely covered with tiny papillae and nearly naked. Scape is as long as two subsequent segments together. The first two segments of club of antennae are transverse, rounded on sides (Figure 7B). Eyes are hemispherical, clearly protruding beyond the head outline. Pronotum is slightly wider than long, its sides rounded; it is the widest in the middle length. Anterior margin of the pronotum is deeply arched, on the sides ending with hook-like, forward-pointing denticles. Most of the pronotum surface is covered with grooves of various sizes. Posterior part of the pronotum is covered with thick papillae. A smooth line running from the middle of posterior margin of the pronotum to its apex is always clearly visible all along. Anterior angles of the pronotum are clear in the form of triangular calli, more or less sharpened. Setation of the pronotum is composed of narrow, recumbent, golden setae, which form larger groups on posterior corners, in the top part, and in the middle of the posterior margin of pronotum. Scutellum highly convex, clearly protruding above the surface of elytra, covered with recumbent, golden setae. Humeral protuberances are poorly pronounced. Parascutellar carinae are clearly visible, shining, strongly protruding above the surface of elytra. Elytral puncturation is chaotic, punctures are deep and densely located. The areas between punctures are narrow, clearly narrower than the puncture diameters, shining and covered with tiny, flattened papillae. Elytral setae are narrow, golden, forming oval patches, and well-isolated from each other. Elytra are jointly sharpened at apices (Figure 7C). Posterior margins of elytra are slightly thickened. The connections of apical margins of elytra and lateral margins of declivity are not clear. Elytral margins are smooth, not crenated on the ventral edges. The ventral side of the body is red–tawny. Central suture of the metasternum is a narrow, dark line that disappears just before the anterior process of the metasternum. Intercoxal process of the first abdominal sternite as in Figure 7D,E. Tibia in their top parts are slightly more densely covered with golden setae than on the remaining area.

Biology and phenology: it is only known to fly to sources of artificial light.

It was described from Andamans [15]. In the same year, it was described under the name *Bostrychus khmerensis* from Cambodia [16]. The latter one was synonymized in the *World’s Catalogue of Bostrichidae* [2].

**Figure 8:**
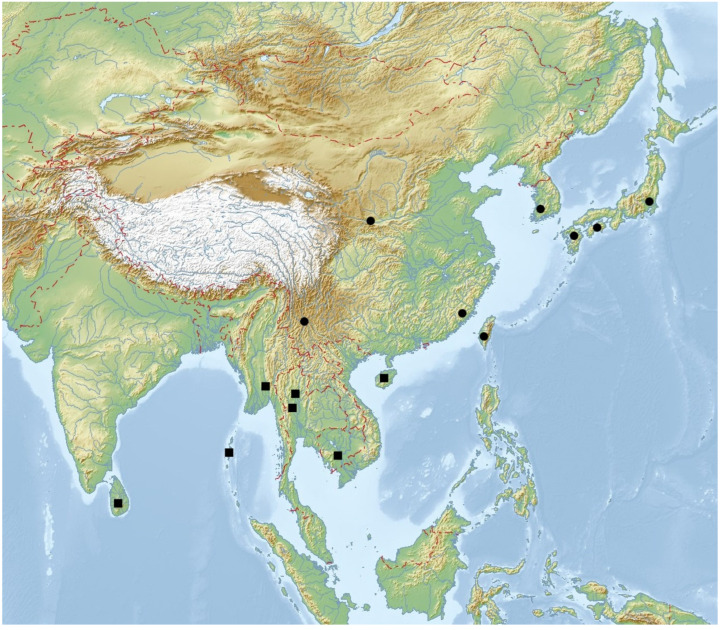
Geographical distribution of *Lichenophanes* species in East and South Asia; square—*Lichenophanes carinatus* (Lewis); dot—*Lichenophanes carinipennis* (Lewis).

Distribution: Andamans, Sri Lanka, Cambodia, Myanmar, Thailand, and China (Hainan Is.) (Figure 8).

### 3.6. Description, Biology, and Geographical Distribution of Lichenophanes carinipennis (Lewis, 1896) (Figure 9A)

*Apate carinipennis* Lewis, 1896: 338 [15]
*Bostrychus guttatus* Matsumura, 1915: 129 [17]

**Figure 9:**
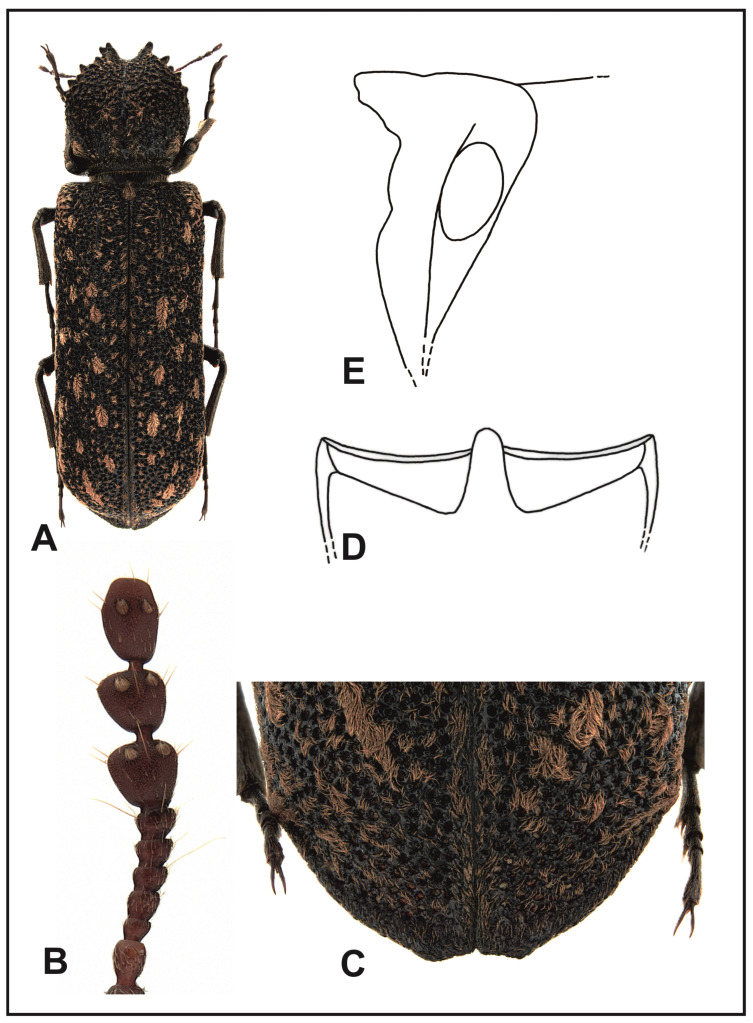
*Lichenophanes carinipennis* (Lewis): (**A**) body, dorsal view; (**B**) antenna; (**C**) apex of elytra; (**D**) intercoxal processes of the first abdominal sternite, dorsal view; (**E**) intercoxal processes of the first abdominal sternite, lateral view.

Description: Body length, 8–16 mm. Body is cylindrical, elongated, black–tawny. Anterior margin of labrum is clearly arched, with densely spaced, golden setae. Anterior margin of clypeus is shallowly arched. Clypeus covered with slightly raised, densely spaced, yellow–gray setae. The suture separating the clypeus from frons is clearly visible only in its central part. Frons with papillae is densely covered with golden, recumbent setae. The vertex and occiput are covered with tiny papillae, nearly naked. The scape of antennae is as long as the three subsequent segments together. The first two segments of the club of antennae are of trapezoid shape, with straight sides (Figure 9B). Eyes are hemispherical, clearly protruding beyond the outline of head. Pronotum is slightly wider than long. Pronotum sides are rounded; pronotum is the widest in the middle length. Anterior margin of the pronotum is narrowly but deeply dentated, ending with hook-like, forward-pointing denticles on sides. A major part of the pronotum is covered with ridges and grooves of various sizes. The middle of the posterior part of the pronotum is covered with thick papillae. A smooth line running from the middle of posterior margin of the pronotum to its apex is invisible, or visible only in a very short distance, in the apical part of pronotum. Anterior angles of the pronotum are clear, in the form of bluntly ended calli. Pronotum setation is composed of narrow, recumbent, golden setae, which form larger groups in posterior angles, in the apical part, and in the middle of the posterior margin of the pronotum. Mesoternal scutellum is strongly convex, clearly protruding above surfaces of elytra, covered with recumbent, golden setae. Humeral protuberances are poorly exposed. Parascutellar carinae of elytra are clear, shining, and strongly protruding over elytra surface. Elytral punctures are chaotically distributed, deep, and densely spaced. Areas between elytral punctures are at least as wide as the puncture diameter or wider, shining, covered with tiny papillae. Elytral setae are narrow, golden, forming oval patches, and well-isolated from each other. Elytral apices with joint declivity (Figure 9C). Posterior elytral margins are slightly thickened. The connection of elytral surface margins and parallel margins of declivity is not clear. Elytral margins on the ventral edges are smooth, not crenated. Ventral part of the body is black–tawny. Central suture of the metasternum is in the form of a narrow line, which disappears before the anterior metasternum process. Intercoxal process of the first abdominal sternite as in Figure 9D,E. Tibia in their top parts are slightly more densely covered with golden setae than on the remaining area.

Biology and phenology: Poorly known. Adults are active at night and fly to sources of artificial light. In Taiwan, adults have been reported to swarm on the trunks of dead standing trees partly devoid of bark (original information).

It has been described from Japan, in the locality of Kawatchi [15,18]. As there are several places named Kawachi (=Kawatchi), it is difficult to clearly determine which of them is *locum typicum*. Lesne, perhaps after contacting Lewis, in his paper of 1901 [19], added the following information below the species: “Kawatchi, côte nord de Nippon”. However, the labels below the holotype (MHNUK) lack such information and read only “Kawachi, Japan” and “Kawatchi, 1874”. In 1915, the species was described again as *Bostrychus guttatus* from Kumamoto-Prefecture [17], located on Kyushu island. Then, the species was synonymized by Chûjô in 1936 [20].

Distribution: China (Yunnan, Fujian, and Shaanxi), South Korea, Japan, and Taiwan (Figure 8).

## 4. Keys for Identifications

### 4.1. Key for the Identification of Western-Palaearctic Species of the Genus Lichenophanes

1. Surface of elytra is covered with recumbent yellow, yellow–gray or golden setae, which form a marbled pattern.………………………………………………………………………………………………………………………2.

- Surface of elytra almost naked; scarce, hardly noticeable, short, and recumbent setae on the sides and margins of elytra, and also in the area of elytral declivity…………………………………………………..*L. juxtaorientalis* n. sp.

2. Intercoxal process of the first abdominal sternite, conical in shape, evenly narrowing toward the apex, where it is narrowly rounded (Figure 6B); apical margins of elytra are slightly flattened and elongated toward the back……………………………………………………………………………………………..*L. numida* (Lesne).

- Intercoxal process of the first abdominal sternite with partly parallel sides, widely rounded at the top (Figure 3B and Figure 5B); apical margins of elytra are not flattened and not elongated toward the back.……………………….3.

3. Surface of elytra is between punctures with tiny papillae; setae of elytra are dense and form numerous, wide patches that occur over nearly all their area……………………………………………….*L. varius varius* (Illiger).

- Surface of elytra is between punctures without tiny papillae; setae of elytra form small, rarely distributed patches; the size and layout of patches resemble *L. numida* more than *L. varius……*..……*L. varius turanicus* n. subsp.

### 4.2. Key for the Identification of Eastern Asian Species of the Genus Lichenophanes

1. Elytra at apices jointly sharpened (Figure 7C). Scape is as long as two subsequent segments. The first two segments of the club of antennae are rounded on the sides (Figure 7B). Areas between elytral punctures are narrow, and clearly narrower than the puncture diameter………………………………………*L. carinatus* (Lewis).

- Elytral apices with short but clear declivity (Figure 9C). Scape is as long as the three subsequent segments. The first two segments of the club are straight on the sides (Figure 9B). Areas between elytral punctures are at least as wide as the puncture diameter or wider……………………………………………………….*L. carinipennis* (Lewis).

## Data Availability

Materials used in this work are available in the collections of the Department of Forest Protection, SGGW, Warsaw, Poland.
